# Convection Parameters from Remote Sensing Observations over the Southern Great Plains

**DOI:** 10.3390/s25134163

**Published:** 2025-07-04

**Authors:** Kylie Hoffman, Belay Demoz

**Affiliations:** Department of Physics, University of Maryland, Baltimore County (UMBC), Baltimore, MD 21250, USA; bdemoz@umbc.edu

**Keywords:** convection, CAPE, CIN, boundary layer

## Abstract

Convective Available Potential Energy (CAPE) and Convective Inhibition (CIN), commonly used measures of the instability and inhibition within a vertical column of the atmosphere, serve as a proxy for estimating convection potential and updraft strength for an air parcel. In operational forecasting, CAPE and CIN are typically derived from radiosonde thermodynamic profiles, launched only twice daily, and supplemented by model-simulated equivalent values. This study uses remote sensing observations to derive CAPE and CIN from continuous data, expanding upon previous research by evaluating the performance of both passive and active profiling systems’ CAPE/CIN against in situ radiosonde CAPE/CIN. CAPE and CIN values are calculated from Atmospheric Emitted Radiance Interferometer (AERI), Microwave Radiometer (MWR), Raman LiDAR, and Differential Absorption LiDAR (DIAL) systems. Among passive sensors, results show significantly greater accuracy in CAPE and CIN from AERI than MWR. Incorporating water vapor profiles from active LiDAR systems further improves CAPE values when compared to radiosonde data, although the impact on CIN is less significant. Beyond the direct capability of calculating CAPE, this approach enables evaluation of the various relationships between the water vapor mixing ratio, CAPE, cloud development, and moisture transport.

## 1. Introduction

In recent decades, the increased presence of ground-based active and passive remote sensing tools has allowed scientists to profile the lower troposphere, known as the planetary boundary layer (PBL), at high temporal and spatial resolutions and to observe processes that occur within the PBL on these smaller scales. Weckwerth et al. [1] and references therein provide one example of this, where data from the Plains Elevated Convection At Night (PECAN) field campaign were used to categorize convection events using radar Plan Position Indicators (PPI) composites. These events were classified into five main types: frontal overrunning, low-level jet only, near mesoscale convective system (MCS), bore/density current, and pristine. The authors found the most substantial percent of cases examined were triggered near an MCS (35%), often associated with elevated heat and moisture transport by a low-level jet, or by a bore and/or density current (25%). A combined 60% convection related to the redistribution of temperature from an MCS/bore, mostly propagating as density currents, is substantial and requires a detailed view of vertical thermodynamics. At night, cold pool, and related density currents are ubiquitous and have been related to many atmospheric phenomena ranging from convection initiation, radiation, dust, and even distribution of insects [2] and references therein. These conditions are mostly shallow and require a high vertical profiling capability to be accurately sampled. Past studies of cold-pool modeling [3] have recommended high-detail horizontal (vertical) grid spacings of 100 m (50 m) or finer to capture the physics of turbulent processes. Coarser resolutions [4] lead to weaker dynamics and thus convective initiation.

Plan position indicator scans of radar reflectivity, used in [1] for classification, provide valuable information about the spatio-temporal evolution of mesoscale structures during convection initiation (CI) but lack important thermodynamic information on the vertical stratification. Conversely, continuous vertical profilers, in particular those that provide temperature, water vapor, and wind, allow for deeper exploration into the vertical structure of forcing mechanisms and the precise elevations associated with energy transport throughout convection initiation processes. As a result, these surface-based profilers have been used for decades to characterize PBL structures [5,6,7,8]. More recently, Grasmick et al. [9] demonstrated a method for calculating Convection Available Potential Energy (CAPE) and Convective Inhibition (CIN) using surface-based profile data in their observational study of the 15 July 2015 PECAN event, although their study was limited to passive AERI observations. In this study, we extend their analysis to many other types of currently available commercial and research-grade remote sensors used in PECAN.

During the PECAN field campaign, in addition to AERI, several other remote sensing tools (Table 1) collected continuous observations, including Raman LiDAR (RL) and Differential Absorption LiDAR (DIAL) water vapor profiling systems, which actively profile the lower atmosphere, and Microwave Radiometer (MWR), which passively profiles the atmosphere. These systems produce higher vertical resolution observations (~30 m), which provide detailed vertical cross sections of rapidly evolving boundaries, waves, and other PBL features.

Our analysis is motivated by questions posed by the 2018 NASA Decadal Survey for Earth Science and Applications [17] and a subsequent workshop report [18]. These reports bring about the central question of the PBL’s influence on convection and air quality and the need for observing the PBL with high vertical and horizontal/temporal resolution, a component particularly relevant to this work. The PBL Incubation Report [18] suggests horizontal, vertical, and temporal measurement requirements for atmospheric profiling (see Table 2). The focus of the table is primarily for temperature and moisture profiling within the PBL, and objectives are labeled as either important (I), very important (VI), or most important (MI). A more detailed discussion of PBL observing systems and measurement requirements is given in the full PBL Incubation Report [18].

As the adoption of remote sensing tools continues to grow and the technology evolves, an accurate appraisal of these measurements’ performance in the derivation of complex data products, such as CAPE/CIN, is important. Instrument data products may incorporate climatological data to append shortcomings and/or use merged datasets from co-located instruments, diffusing the “true” capability of the basic physics-based retrieval data product. We demonstrate an example by using data from the PECAN field campaign during the 14–15 July 2015 MCS events, both from passive (AERI, MWR) and active (DIAL, RL) sensors, as well as their accuracy in calculating CAPE and CIN when compared to radiosonde data as truth. The effects of water vapor content and vertical distribution of water vapor in the PBL are discussed in terms of their impact on convection potential and moisture redistribution within the PBL. We also conduct an instrument resolution analysis and an evaluation of the Weather Research and Forecasting (WRF) model in calculating CAPE and CIN for these events.

This paper is organized as follows: Section 2 describes the water vapor LiDAR systems used in the PECAN field campaign and the method used for calculating CAPE/CIN with the observational datasets. Section 3 discusses the synoptic and surface meteorology setup for the cases considered here. CAPE/CIN values calculated from remote sensing profilers are also shown in this section. Section 4 provides a summary and discussion of the results.

## 2. Materials and Methods

### 2.1. Water Vapor LiDARs: MPD, LASE, and ALVICE

**Figure 1:**
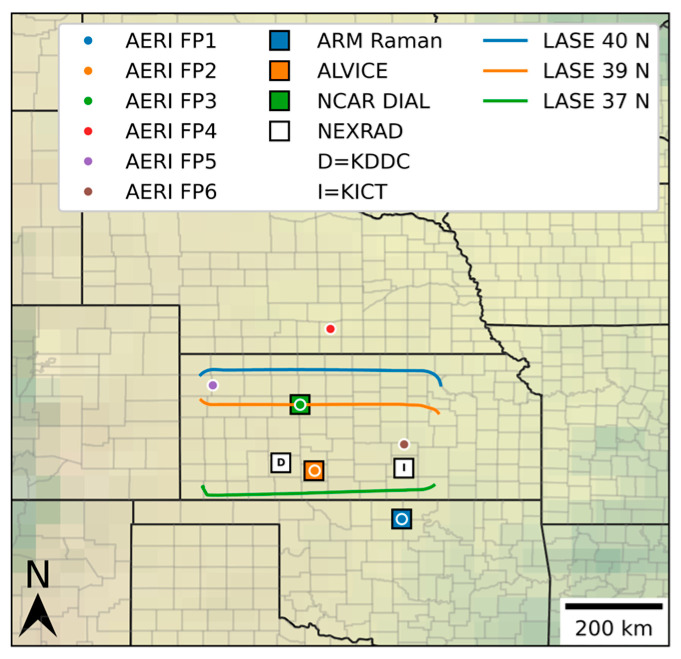
Locations of AERI and LiDAR systems in the SGP region during the PECAN field campaign. Small dots represent AERI systems, and large squares represent ALVICE, Raman, and DIAL LiDAR systems. Flight paths corresponding to airborne LASE missions are shown in blue (40° N), orange (39° N), and green (37° N).

Water vapor LiDARs were stationed at ARM SGP sites FP2 and FP3 (Table 1; Figure 1) during the PECAN campaign. MicroPulse Differential Absorption LiDARs (NSF-NCAR, Boulder, CO, USA; MPD) have been reported extensively in the literature [1,13,19,20] and were located at FP3 during these cases. At FP2, the Atmospheric LiDAR for Validation, Interagency Collaboration, and Education (ALVICE) LiDAR (NASA-GSFC, Greenbelt, MD, USA), a Raman LiDAR system, was operational during PECAN as well. Details of that system and measurement capability are extensively discussed in Whiteman et al. [15]. Further, the airborne DIAL system called the LiDAR Atmospheric Sensing Experiment (NASA-LaRC, Hampton, VA, USA; LASE) was used for horizontal variability of the water vapor during these cases. LASE has been extensively used and reported in the literature [12,21,22].

**Figure A1:**
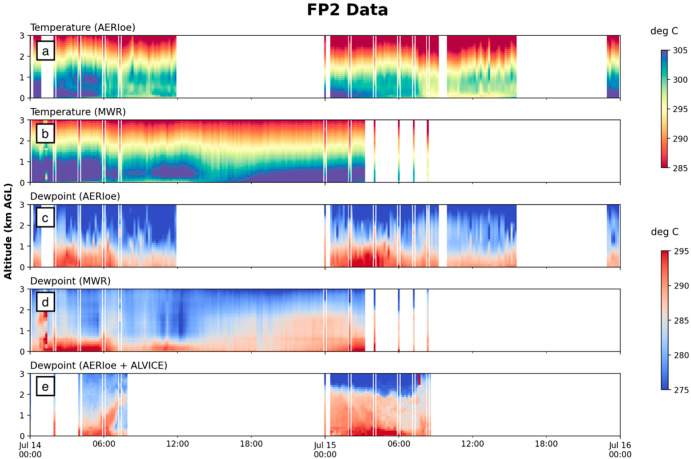
Data collected by remote sensors at SGP site FP2. Temperatures from AERI (**a**) and MWR (**b**) are shown, followed by dewpoint temperatures from AERI (**c**), MWR (**d**), and ALVICE (**e**). Vertical “lines” are radiosonde data for reference.

**Figure A2:**
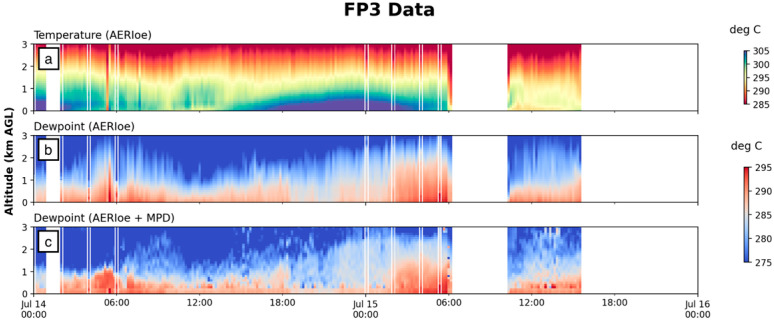
Data collected by remote sensors at SGP site FP3. Temperatures from AERI (**a**) are shown, followed by dewpoint temperatures from AERI (**b**) and MPD (**c**). Vertical “lines” are radiosonde data for reference.

These systems actively profiled water vapor content through the PBL and some of the free troposphere using Raman and Differential Absorption (DIAL) techniques. Many of the systems do not observe temperature profiles; therefore, this study combines AERI-retrieved temperature profiles with LiDAR-observed water vapor to calculate CAPE (see Table 3 for details). Further, water vapor profile information above the highest range of LiDARs was filled in with AERI water vapor values as well to allow for integration of the column in CAPE/CIN calculations. Appendix A, Figure A1 and Figure A2 show the AERI-retrieved temperature profiles and merged AERI + LiDAR water vapor profile products used in these calculations.

### 2.2. CAPE Methodology

In order to generate complete and continuous time–height cross sections of CAPE and CIN, values were calculated for each observation time and altitude of available remote sensing data on 14–15 July 2015. AERI (University of Wisconsin Space Science and Engineering Center, Madison, WI, USA; FP2, FP3) and MWR (Radiometrics, Frederick, CO, USA; FP2) datasets each contained temperature and moisture variables; thus, CAPE/CIN were calculated for these instruments independently. Surface-based RL (FP2) and MicroPulse DIAL (MPD; FP3) provided water vapor vertical profiles, although these systems lack temperature data; therefore, collocated AERI temperatures were used to calculate CAPE/CIN (Table 3). The airborne water vapor DIAL, part of the LiDAR Atmospheric Sensing Experiment (LASE), was used in a similar manner to calculate CAPE in combination with temperature profiles from ERA5 reanalysis data, a global reanalysis dataset from the European Centre for Medium-Range Weather Forecasts, with hourly data and 31 km horizontal resolution [23]. The locations of each instrument and the flight path of LASE on 14 July are shown in Figure 1 below.

Temperature, pressure, and dewpoint temperature data were used to calculate CAPE and CIN from the datasets. In some cases, dewpoint temperature was not reported directly, and other moisture variables were used to determine dewpoint temperature (see Table 3). For each observation time and altitude, the corresponding parcel’s thermodynamic pathway was calculated to determine its lifting condensation level (LCL), level of free convection (LFC), and equilibrium level (EL). In cases when a parcel did not reach an EL, the top of the observation profile is used as the EL, and the dataset is flagged as such. CAPE and CIN were then integrated over the parcel’s path as follows:

$$CIN=-R_{d}\sum_{surface}^{LFC}T_{parcel}-T_{environment}d\;ln\;\left(P\right)\;,$$

(1) $$CAPE=-R_{d}\sum_{LFC}^{EL}T_{parcel}-T_{environment}d\;ln\;\left(P\right)\;.$$

## 3. Results

### 3.1. Case Overview: Surface Meteorology, Synoptic Setting, and Radar Description

The case studies selected are two consecutive days during the PECAN field campaign, 14 and 15 July 2015, which saw similar synoptic dynamic setups (nearby surface low pressure, 500 mb ridge) and convection triggering mechanisms (a series of bore waves coupled with existing MCS cold pools). Atmospheric bores are a frequently observed atmospheric disturbance in the form of an abrupt pressure jump. They are often initiated along the leading edge of a cold pool (the gust front) in statically stable atmospheres and can propagate hundreds of kilometers, triggering new convection via vertical lifting [24]. Bores are accompanied by a rise in pressure and shift in surface wind [25,26], allowing for identification from in situ surface observations Although the two days considered here were forecast to have CI over the intensive observation area around FP2, and bore waves were observed on both of these days, only one of them (15 July) resulted in convection. Details of the bore and other aspects of these cases are discussed elsewhere [9,27,28].

**Figure 2:**
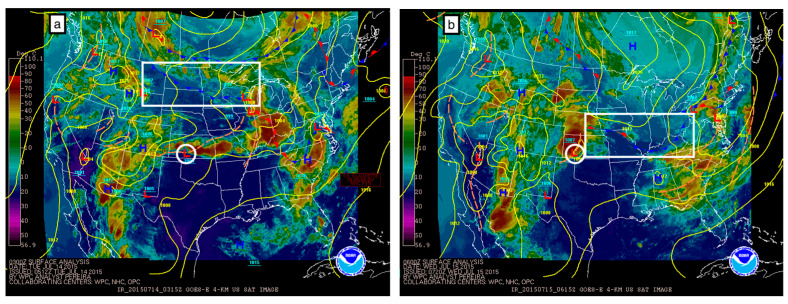
Synoptic meteorology maps of the contiguous United States generated by NOAA on (**a**) 14 July 2015 at 03:15 UTC and (**b**) 15 July 2015 at 06:15 UTC. Color-filled contours indicate GOES-East cloud top temperatures and correspond to values on the left-side colorbar. Surface pressure isobars are shown as solid yellow lines. Surface frontal boundaries are demarcated using traditional frontal boundary symbols (blue triangles, red half-moons), and low-/high-pressure centers are marked by red L and blue H labels, respectively. Features of interest are identified by white annotations, including a cold front (white rectangle) and low-pressure center (white circle) located in the central US.

Despite identical synoptic settings and trigger mechanisms (bore activity), the two case studies resulted in different forecast outcomes. In this section, we first describe the synoptic setting for the cases below, followed by a report on CAPE and CIN evolution with respect to instrument type and vertical resolution. The synoptic setting for the two consecutive studies was similar in formation, and no appreciable synoptic change was observed. On the night of 14 July, a region of surface low pressure was present over southwest Kansas (Figure 2a, white circle annotation), with an associated surface trough extending to its southwest and northeast. A large frontal system was passing through the Midwest and mid-Atlantic region (Figure 2a,b, white rectangle annotation). Over a period of 27 h, the Kansas low pressure traveled east to Tennessee, and the cold front associated with the midwestern frontal system shifted to the central plains, allowing for a new low pressure and surface trough to form over southwest Kansas (Figure 2b, white circle annotation). At the 500 mb level a ridge pattern persisted over the southern plains region, and its structure and positioning did not change significantly. The rapid cold frontal movement, development of a similar surface low-pressure center over the Kansas-Oklahoma border, and persistence of the 500 mb layer ridge resulted in very similar synoptic dynamic conditions on the nights of 14 and 15 July. The only difference between these two days is the duration after the passage of the frontal system.

**Figure 3:**
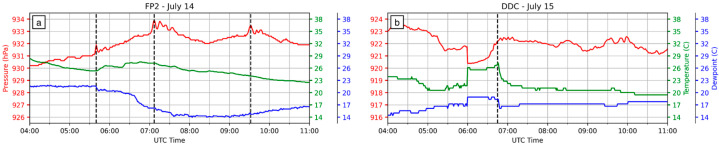
Surface meteorology time series data for pressure (red), temperature (green), and dew point temperature (blue) on (**a**) 14 July (measured at FP2) and (**b**) 15 July (measured at DDC). The times of four bore wave overpasses (July 14: 5:40 UTC, 7:07 UTC, 9:32 UTC; July 15: 6:45 UTC) are noted by black dashed vertical lines.

Surface meteorology observations (Figure 3a) show spikes in pressure associated with three bore wave overpasses at FP2 on the night of 14 July: the first at 5:40 UTC, the second at 7:07 UTC, and the third at 9:32 UTC. Temperature and dewpoint shifts are most pronounced during the first bore wave, less distinct but still present during the second bore wave, and absent during the third bore wave. Surface meteorology was not recorded at FP2 on 15 July, although data from the nearby Dodge City (DDC) ASOS station is shown in Figure 3b. Strong pressure, temperature, and dewpoint signatures are recorded at 6:45 UTC on 15 July as a bore passed over DDC.

**Figure 4:**
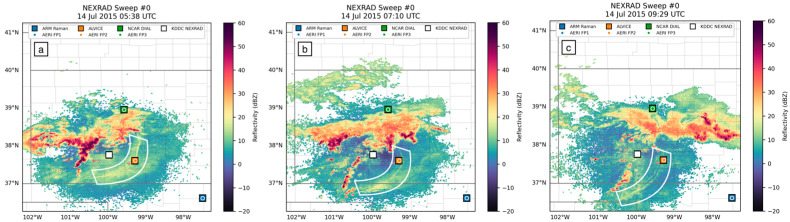
NEXRAD radar reflectivity showing three bore waves passing over FP2 on 14 July at (**a**) 5:40 UTC; (**b**) 7:07 UTC; and (**c**) 9:32 UTC. Bore waves are outlined by white meniscus annotations.

The MCSs on these two nights can be seen in radar reflectivity images as well (Figure 4), represented by orange, red, and purple areas (30–60 dBZ). Their respective bore waves propagating to the southeast are annotated by white meniscus shapes, and resulting new convection is annotated by red meniscus shapes. Three bore waves were indicated to have passed over FP2 on 14 July, indicated by surface pressure readings and WSR-88D NEXRAD radar reflectivity images. None of these three bore waves led to the generation of new convection along their boundaries, as seen in Figure 4a–c.

**Figure 5:**
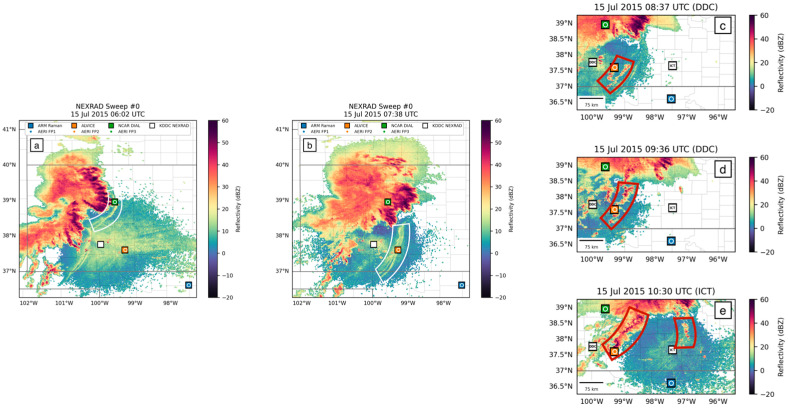
NEXRAD radar reflectivity showing two bore waves passing on 15 July: (**a**) 6:02 UTC at FP3; (**b**) FP2 7:38 UTC. Bore waves are outlined by white meniscus annotations. New convection resulting from the second bore are outlined by red meniscus annotations roughly (**c**) one hour; (**d**) two hours; and (**e**) three hours after initiation.

The following night, on 15 July, the first bore wave passed over FP3 but quickly dissipated and did not trigger any new convection (Figure 5a). The second bore on 15 July, which passed over DDC at 6:45 UTC and then FP2 at 7:38 UTC (Figure 5b), did lead to convection initiation beginning at 7:45 UTC (Figure 5c). One hour later, an organized line of new cells, approximately 100 km long, had formed to the south of FP2, and within the following hour these cells strengthened into a line of storms approximately 135 km long (Figure 5d).

**Figure 6:**
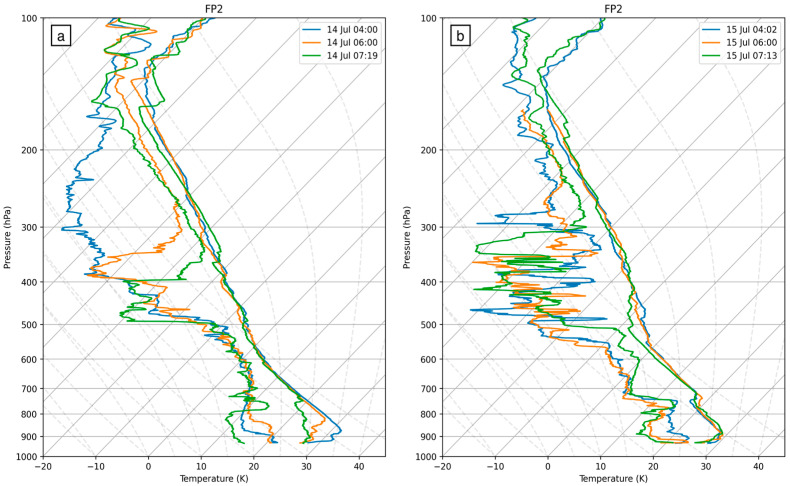
Radiosonde composites from FP2 of temperature and dew point temperature (temperature > dewpoint) at approximately 4:00 UTC (blue), 6:00 UTC (orange), and 7:15 UTC (green) on (**a**) 14 July and (**b**) 15 July.

Radiosonde profiles (Figure 6) indicate an extremely dry upper layer (200–300 hPa) at 4:00 UTC on 14 July, as well as a warm PBL—a large contrast to the moist upper atmosphere and cool boundary layer found at the same time on the following day. On 14 July, from 4:00 UTC to 6:00 UTC, the dry upper layer significantly moistened as the MCS strengthened and approached the observation sites. During this time period, the PBL began to progressively cool, eroding the stable surface layer while dew point temperatures rose, which led to notable moistening in the first few kilometers. However, dew point temperatures fell five degrees in just over an hour after 6:00 UTC, leading to significant drying of the layer aligning with the MCS’s exit from the region. On 15 July, the upper layer was already fairly moist leading up to the MCS approaching the SGP sites. The surface layer remained very stable on this day, while dew point temperatures slightly dropped in the boundary layer from 4:00 to 6:00 UTC. In general, the radiosonde profile, as in the synoptic setup discussed above, shows a similar upper air temperature profile but an evolving and increasing moisture, both within and above the PBL, allowing us to consider the effect of the moisture on CAPE and CIN.

### 3.2. Traditional CAPE and CIN Methods

**Figure 7:**
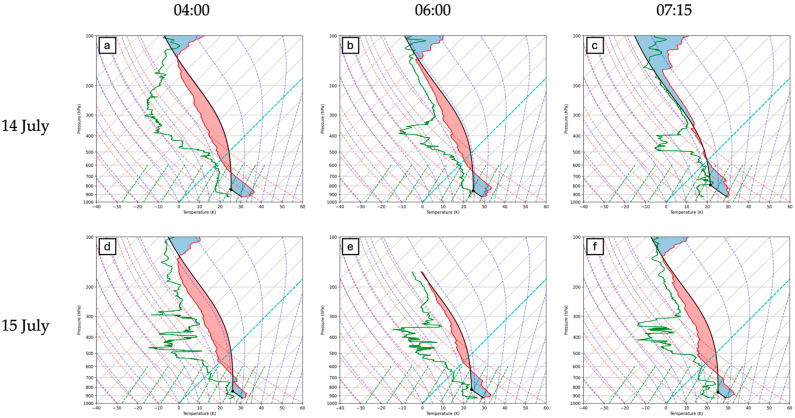
Skew-T Log-P diagram of radiosonde thermodynamic profiles at approximately 04:00 UTC, 6:00 UTC, and 07:15 UTC on (**a**–**c**) 14 July and (**d**–**f**) 15 July. Surface-based CAPE is shaded in red and surface-based CIN is shaded in blue.

**Figure 8:**
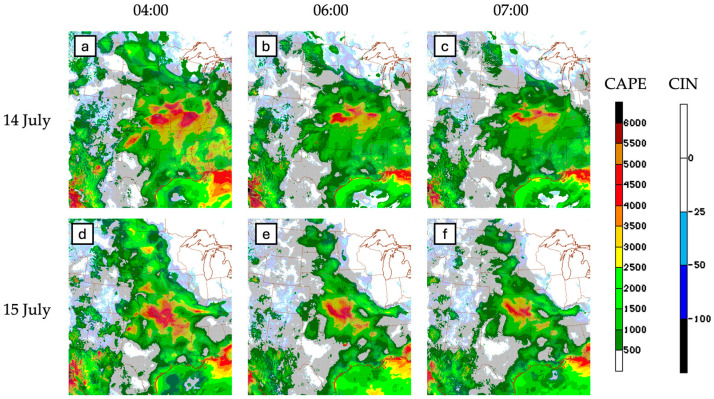
HRRR surface-based CAPE (jet colors) and CIN (blue) at 4:00 UTC, 6:00 UTC, and 7:00 UTC on 14 July (**a**–**c**) and 15 July (**d**–**f**).

The standard way of looking at the evolution of surface-based CAPE is shown in Figure 7 (from radiosonde) and Figure 8 (from High Resolution Rapid Refresh; HRRR). The magnitude of regional surface-based CAPE on these two days is similar. However, the HRRR CAPE shows the region of highest surface CAPE farther south on 15 July due to the proximity of the cold front on this day, which acts to restrict transport of heat and moisture farther north into the central plains. Since the synoptic conditions (ridge pattern, surface low pressure) and triggering mechanism (bore wave) are similar on the two days, we contend that other factors, including the presence of elevated moisture and the proximity of the cold front, are primary controls of the moisture evolution and vertical distribution leading up to the trigger and two MCS events.

Radiosonde measurements provide valuable insight into the vertical column, although the 1–2 h temporal frequency shown in Figure 7 is rare and reserved for intensive campaigns such as PECAN. Operational radiosonde launches occur twice daily at 00:00 UTC and 12:00 UTC. Model-simulated CAPE/CIN (Figure 8) provides more insight into the temporal and spatial evolution of CAPE/CIN; however, these are model approximations and are not based off observed values. Continuous ground-based remote sensing fills part of this radiosonde–model gap by providing continuous time–height cross sections. The sections to follow show remote sensing CAPE and CIN values for the 14 and 15 July cases, derived from AERI, MWR, water vapor LiDARs (ALVICE, MPD), and airborne LASE DIAL in the sections below. A statistical comparison between radiosonde-derived and remote sensing-derived CAPE and CIN is provided for each instrument. Radiosonde data is used as “truth” data in evaluations of remote sensing accuracy.

### 3.3. Passive Remote Sensors: AERI and MWR

Two passive remote sensing instruments, AERI [10] and MWR [16], were deployed at several of the ARM SGP sites during PECAN. A brief analysis of CAPE and CIN values calculated from data at the FP2 location below compares the two passive sensors’ accuracy in calculating CAPE and CIN when compared to radiosonde data as the ground truth. Temperature and dewpoint temperature values used for these calculations are shown in Appendix A.

**Figure 9:**
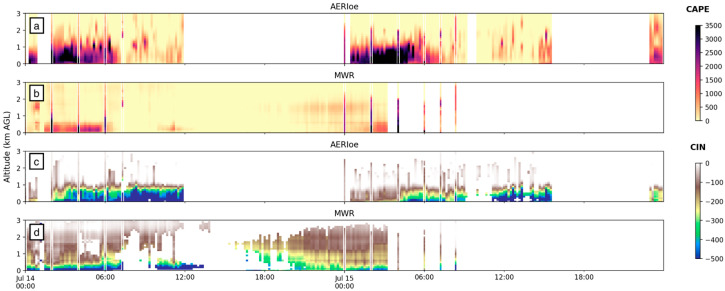
CAPE (**a**,**b**) and CIN (**c**,**d**) values for all available data collected by AERI and MWR systems, respectively, at FP2 on 14–15 July 2015. Vertical “lines” are derived from radiosonde data and plotted using the same range of shading for CAPE and CIN, shown on the side.

Figure 9 shows time–height cross sections of CAPE calculated from AERI and MWR with vertical lines of radiosonde-derived CAPE for reference. A quick glance clearly reveals that MWR significantly underestimates (overestimates) CAPE (CIN) for most of the profiled atmosphere on both 14 and 15 July. AERI-derived values are relatively closer to the radiosonde and perform better in estimating CAPE and CIN within the first 1–2 km and perform better overall on 14 July. However, on 15 July, AERI-derived CAPE fell to near-zero values between 1 and 2 km, while radiosonde-derived CAPE remained high up to about 2 km. Since the temperature evolution and synoptic setting for both these days were similar, we contend that most of the variation should be accounted for by variation in moisture content. The profile-to-profile calculated CAPE shows much more structure similar to the water vapor mixing ratio derived from a Raman LiDAR compared to the MWR, which does not account for or incorporate the actively sensed water vapor information and uses only climatology to derive the coefficients [29]. The CAPE/CIN performance then is dictated by the ability (or inability) to resolve the vertical moisture content.

**Figure 10:**
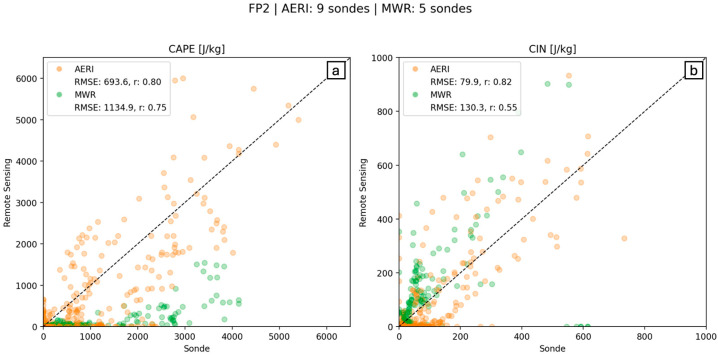
Correlation graph for (**a**) CAPE and (**b**) CIN calculated by AERI data (orange) and MWR data (green; *y*-axis), as compared to radiosonde-derived CAPE (*x*-axis).

The scatter plot correlation graph (Figure 10) compares AERI and MWR to radiosonde CAPE/CIN for the times where data overlaps. AERI-derived CAPE was compared to nine radiosondes over the 2-day period, and MWR-derived CAPE was compared to five radiosondes over the 2-day period. Root Mean Squared Error (RMSE) and Pearson Correlation Coefficient (r) values confirm findings from evaluations of Figure 9 that AERI’s CAPE/CIN values match radiosonde-derived values better than MWR. Figure 10 also highlights the significant underestimation (overestimation) of MWR CAPE (CIN) values, indicating extreme care must be taken when using MWR-derived water vapor profiles for energy forecasts. In fact, all MWR CAPE values from 14 and 15 July are less than radiosonde CAPE values, often by 1000 J/kg or more, a significant underrepresentation.

### 3.4. Active Remote Sensors: MPD and ALVICE

**Figure 11:**
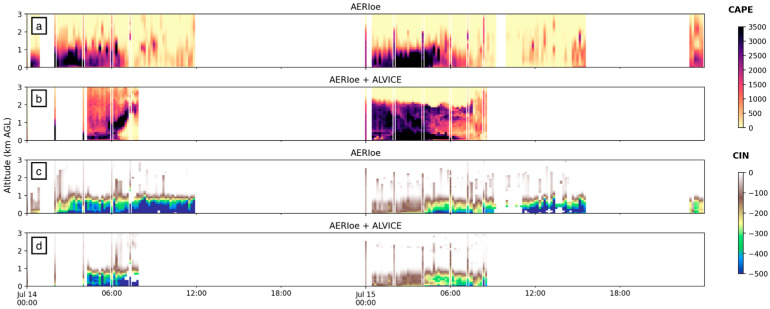
CAPE (**a**,**b**) and CIN (**c**,**d**) values for all available data collected by AERI and AERI + ALVICE systems at FP2. Vertical “lines” are derived from radiosonde data and plotted using the same range of shading for CAPE and CIN, shown on the side.

We compare both the AERI-standalone CAPE and CIN products with the combined AERI + LiDAR CAPE and CIN to estimate the added value from active profiling on CAPE and CIN calculations. The ALVICE LiDAR, located at FP2 and operating in limited duration, performed very well in estimating CAPE and CIN when compared to radiosonde values. Figure 11 shows both AERI standalone (panels a, c) and AERI + ALVICE (panels b, d) CAPE and CIN, respectively. Above the first 1 km, where AERI underpredicted CAPE, ALVICE was very precise in calculating the magnitude and drop-off of high CAPE values. This is true for both days but can be easily seen for all radiosondes on 15 July (Figure 11b), where the radiosonde overlay data appears to blend in seamlessly with the LiDAR curtain. In particular, the CAPE agreement between radiosonde and ALVICE water vapor is an excellent demonstration of the role of the water vapor and the instrument’s capability in resolving the vertical layering in the PBL. CIN estimations produced different results, where AERI-standalone values slightly outperformed AERI + ALVICE values. The RMSE of their respective CIN values differed by only 6.6 J/kg, indicating very similar accuracy overall.

**Figure 12:**
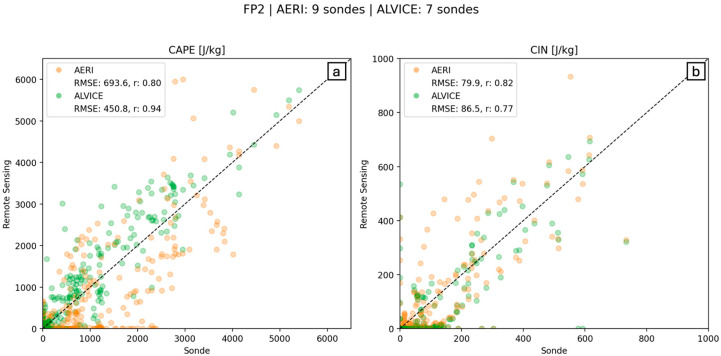
Correlation graph for (**a**) CAPE and (**b**) CIN calculated by AERI data (orange) and AERI + ALVICE data (green; *y*-axis), as compared to radiosonde-derived CAPE (*x*-axis).

Figure 12 shows the same AERI vs. radiosonde correlations as seen in Figure 10 (orange); however, MWR data has now been replaced by ALVICE data (green). The consistent underestimation of CAPE values from AERI-derived data, as can be seen by the high density of orange points along the *x*-axis, is no longer present once ALVICE water vapor mixing ratio data is used. The overall RMSE also drops by 242 J/kg when ALVICE data is added.

**Figure 13:**
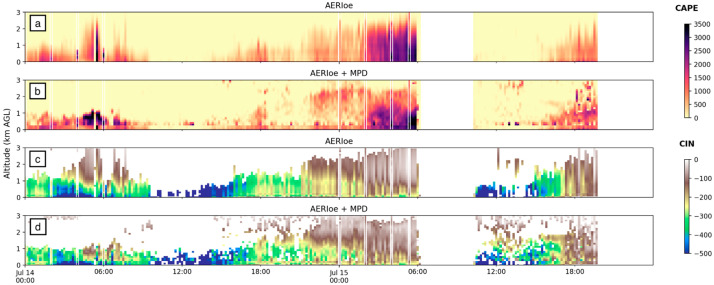
CAPE (**a**,**b**) and CIN (**c**,**d**) values for all available data collected by AERI and AERI + MPD systems at FP3. Vertical “lines” are derived from radiosonde data and plotted using the same range of shading for CAPE and CIN, shown on the side.

**Figure 14:**
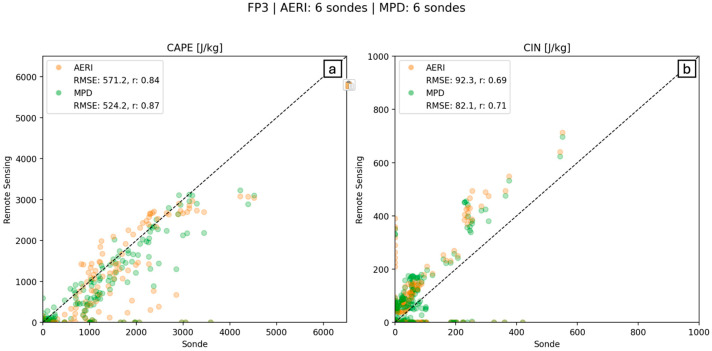
Correlation graph for (**a**) CAPE and (**b**) CIN calculated by AERI data (orange) and AERI + MPD data (green; *y*-axis), as compared to radiosonde-derived CAPE (*x*-axis).

Another water vapor LiDAR system, the MPD [13], was operated at FP3 (the location of the first bore on 15 July). Figure 13 and Figure 14 indicate that the combined AERI + MPD product outperformed the AERI standalone for both CAPE and CIN. Qualitative analyses of Figure 13a,b show that the AERI + MPD product did a better job estimating the magnitude and depth of strong lower-tropospheric CAPE. Figure 14a demonstrates that, on average, AERI + MPD CAPE values are more precise (they fall closer to the 1:1 line than AERI standalone values); however, AERI + MPD slightly underpredicted CAPE on average when compared to radiosonde values. The gains in accuracy from the addition of the ALVICE system were larger than those seen at FP3 from the MPD system, although an advantage of the MPD system is that it performed 24/7 and provided continuous data coverage, more than the ALVICE data sets.

Overall, CAPE values derived using water vapor LiDAR observations are found to be a significantly better match to radiosonde values than passive instruments alone. These results are due to the ability of DIAL and MPD systems to observe the water vapor vertical profile using backscatter signals (and Raman and DIAL techniques), as opposed to passive profiling methods, which rely on retrieval techniques and a priori information to determine water vapor concentrations.

### 3.5. Airborne DIAL: LASE

All the remote sensors we have reported above are fixed at a location and do not give a sense of the spatial variability of the derived products needed for forecasts. Airborne water vapor LiDAR data can mitigate and provide spatial coverage. LASE [12] water vapor profiles on 14 and 15 July show changes in CAPE and CIN estimations along three zonal flight paths. The LASE system did not measure temperature through the column; therefore, ERA5 reanalysis temperature data [23] interpolated to the nearest time, location, and altitude were used to construct vertical profiles of temperature to use for calculations. The assumption is that the temperature variations are minimal, and the CAPE calculations are primarily dictated by water vapor variations, as the ALVICE plot in Figure 11 suggests.

**Figure 15:**
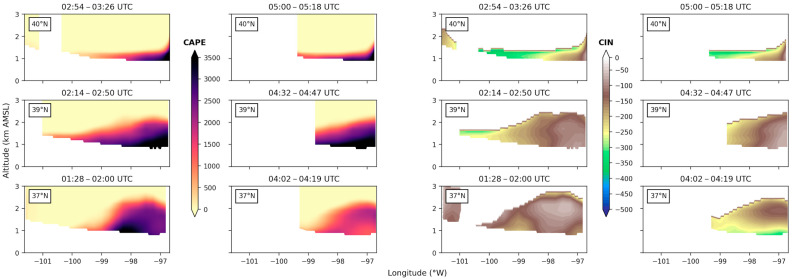
CAPE and CIN from the ERA5 (temperature) and airborne DIAL (water vapor) combined profiles. Three zonal cross sections at 40° N, 39° N, and 37° N (see Figure 8) are shown in each column from top to bottom for each of the two pass-throughs on 14 July.

**Figure 16:**
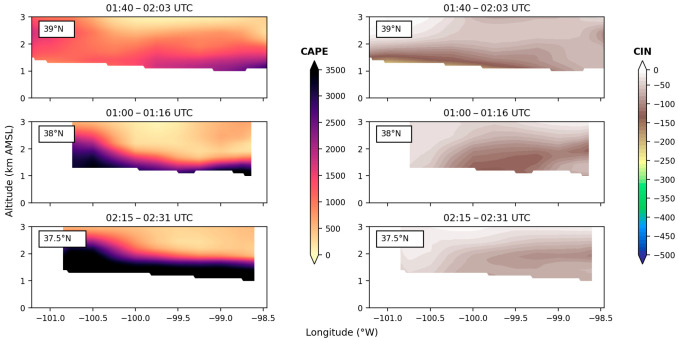
CAPE and CIN from the ERA5 (temperature) and airborne DIAL (water vapor) combined profiles. Three zonal cross sections at 39° N, 38° N, and 37.5° N are shown in each column from top to bottom for a single passthrough on 15 July.

Figure 15 and Figure 16 show CAPE and CIN from the combined ERA5 + LASE products on 14 July (15 July). First impressions of the two figures point to a significant increase in the magnitude of CAPE and a decrease in the magnitude of CIN for the entire 0–3 km column and throughout the entire flight domain between 14 and 15 July. These values indicate the troposphere had less convection inhibition and significantly more potential energy available to aid in the generation of new cell formation on 15 July, conditions that align with NEXRAD observations in Figure 3 and Figure 4. Evaluating 14 July data alone (Figure 15), it is also clear that the magnitude of CAPE decreased between the first and second flyovers of the southernmost region (37° N), which were spaced approximately 2.5 h apart. CIN values increased in magnitude along the 37° N track during this time. However, the magnitude of CAPE and CIN observed along 39° N and 40° N tracks did not change significantly over this period.

CAPE values were very high during the entire flight on 15 July. In particular, the 38° N and 37.5° N tracks show consistently high values of CAPE, exceeding 3500 J/kg in the PBL from 1:00 UTC to 2:30 UTC. This high magnitude of CAPE in the PBL was capped by a deep layer of moderate CAPE ranging from 500 to 1000 J/kg, a sharp contrast to the previous day (Figure 15), where free tropospheric CAPE quickly decreased to near-zero values. Overall, LASE CAPE and CIN values indicate that the SGP’s troposphere had significantly more energy in the form of CAPE and less inhibition in the form of CIN on 15 July than on 14 July. These findings indicate that the atmosphere was primed for convection initiation on 15 July, essentially providing the necessary fuel for new convection initiation along the MCS outflow boundaries.

### 3.6. Model Performance and Towards Water Vapor Assimilation

A companion work has performed extensive analysis of the Weather Research Forecast model’s performance in capturing the water vapor variability observed in these cases and the improvement that can be achieved by assimilation of the radar and LiDAR data sets [28,30]. The Penn State University Weather Research & Forecasting Model Ensemble Kalman Filter data assimilation system (PSU-WRF-EnKF) version 3.8.1 [31], a 3DVAR data assimilation system, was employed in the companion work. Physical parameterization schemes used include the Thompson et al. [32] microphysics scheme, Mellor–Yamada–Janjić turbulence kinetic energy (TKE) scheme for PBL processes, unified Noah land surface model, Monin–Obukhov–Janjić Eta scheme for surface layer parameterization, and the Rapid Radiative Transfer Model for General Circulation Models (RRTMGs) for the longwave and shortwave radiation schemes. The horizontal grid spacings of the parent and nested domains were 3 km and 1 km, respectively, and 61 vertical levels were used, with 19 vertical levels within the lowest 1 km AGL.

Reporting on the details of the performance is beyond the scope of this paper. However, we use simulated data produced with assimilated WSR-88D (RAD) and ALVICE Raman LiDAR [5] (LID) observations at FP2 to evaluate the model’s performance in capturing the CAPE and CIN pre- and post-assimilation to gauge the improvement quantitatively. The focus of this assimilation is on improvements from water vapor observations, although future work incorporating temperature observations would be beneficial. Thus, though not exhaustive, we think the exercise would provide value in model forecast and efficacy of water vapor profile improvement.

**Figure 17:**
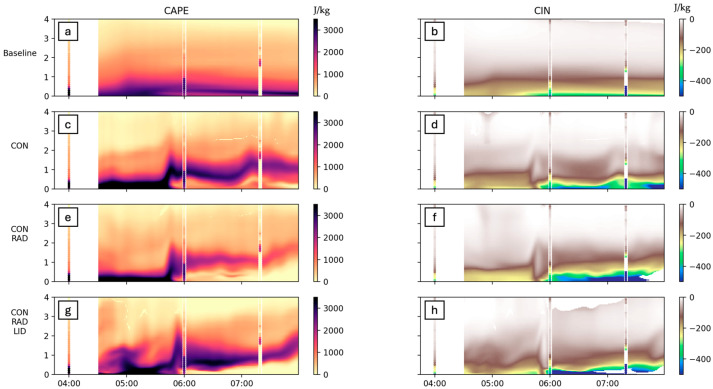
CAPE and CIN calculated by WRF output on 14 July at FP2. Four WRF runs are shown: baseline (**a**,**b**), consensus (**c**,**d**), consensus + radar (**e**,**f**), and consensus + radar + LiDAR (**g**,**h**). Also shown are overlaid radiosonde-calculated values of CAPE and CIN.

PSU-WRF-EnKF, henceforth WRF, simulations of the FP2 site on 14 July show the impact that three data assimilation setups have on CAPE and CIN calculations when compared to a baseline run and to radiosonde-derived CAPE and CIN (Figure 17 overlaid lines). A clear progression in quantitative improvement vis-a-vis the radiosonde values in CAPE from baseline simulation to consensus data assimilation (CON) to CON + RAD and further to CON + RAD + LiDAR is evident. The assimilation of the radar and water vapor profiles also drastically improved the vertical stratification of the water vapor, which was absent in the baseline simulation. However, the altitude where the water vapor was lifted was lower by about 500 m compared to the radiosonde. While the radar assimilation was adequate for the placement of the timing of the initial lift, the moisture assimilation provided some improvement in CAPE values, pre- and post-bore locations.

Comparing WRF (Figure 17) performance to remote sensing values (Figure 11 and Figure 15), WRF tends to estimate CAPE values as greater than remote sensing equivalents. This is particularly true for the radiosonde at 7:15 UTC, where WRF CAPE values range from 0 to 2500 J/kg and radiosonde values range from 0 to 1800 J/kg.

In summary, comparing each data assimilation product discussed here to the radiosonde CAPE and CIN values, the baseline run clearly fails to capture elevated CAPE associated with the outflow boundary and bore wave. Consensus data alone does a good job at introducing the lifting of water vapor and CAPE but lacks some of the detail a remote sensor data assimilation would provide in resolving the wave structure. The addition of radar and LiDAR data further refines the structure of the bore wave, although increasing the amount of data input into the assimilation does not appear to necessarily improve the CAPE accuracy by much.

### 3.7. Vertical Resolution Analysis

**Figure 18:**
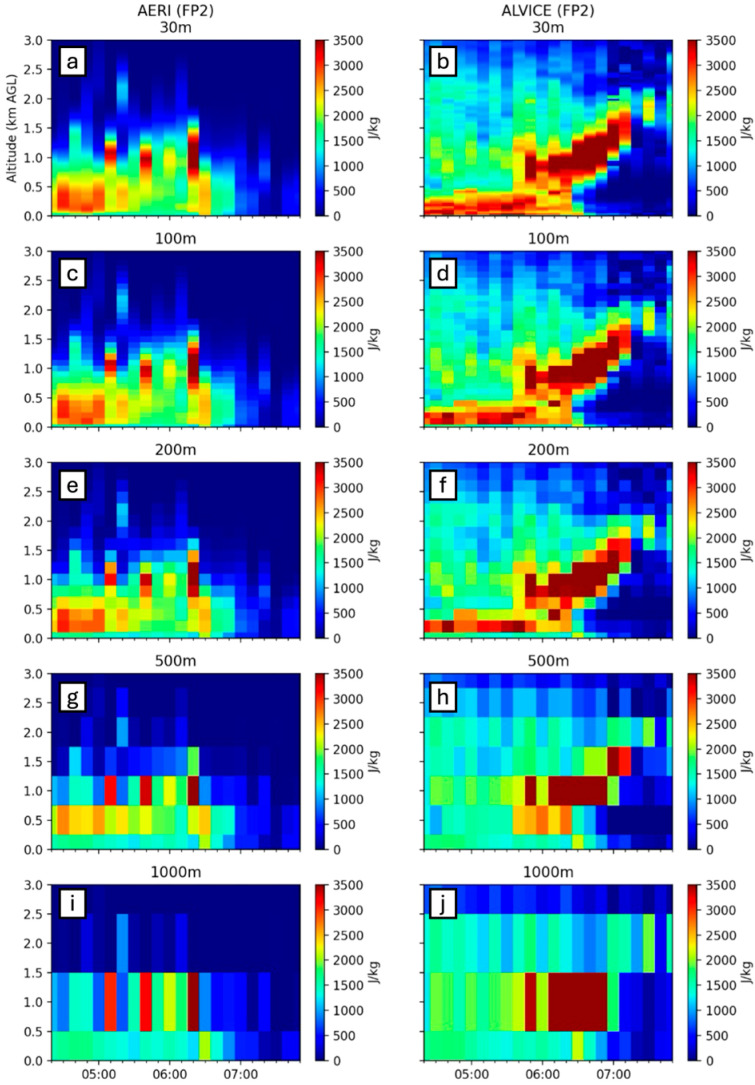
CAPE resolution comparison for AERI and ALVICE at FP2 at resolutions of 30 m (**a**,**b**), 100 m (**c**,**d**), 200 m (**e**,**f**), 500 m (**g**,**h**), and 1000 km (**i**,**j**).

All previous data shown was at a 100 m vertical resolution, coarser than the LiDAR vertical resolutions but finer than the AERI vertical resolution. A brief analysis below shows the impact of data resolution on interpretation of an outflow boundary and region of lifting. Figure 18 shows five vertical resolutions of CAPE from AERI and the AERI + ALVICE data sources at FP2 on 14 July.

The AERI vertical resolution begins at 100 m at the surface and increases with altitude; thus, there is very little difference between the 30 m and 100 m vertical resolutions, despite the ALVICE’s water vapor vertical resolution of 30 m. The impacts of data resolution become more apparent in the transition from 100 m to 200 m and higher, particularly at the surface. The loss of data in the shallow surface layer results in a reduction of surface-based CAPE, most significantly in the 500 m and 1000 m cases. For this reason, vertical resolutions equal to and greater than 500 m are not sufficient for profiling with the intention of calculating PBL convection parameters. A value of 200 m appears to be a well-balanced resolution between ideals from an engineering perspective and a scientific perspective.

## 4. Summary and Conclusion Remarks

This study evaluates various combinations of remote sensing instruments and their effectiveness in estimating convective parameters, Convective Available Potential Energy (CAPE) and Convective Inhibition (CIN), over two days of the PECAN field campaign. Previous studies [9] demonstrated CAPE and CIN calculations from the passive AERI platform. However, here we extend their analysis to include an additional passive profiler (Microwave Radiometer) and several active water vapor profilers (Raman LiDAR, micropulse DIAL, airborne DIAL). Results indicate that active remote sensing systems enhance the accuracy of convection parameters when compared to passive systems alone. These findings underscore the potential of integrated remote sensing platforms to improve our understanding of pre-convective environments and enhance weather forecasting capabilities.

Comparative analysis of passive systems revealed that the Atmospheric Emitted Radiance Interferometer (AERI) outperformed the Microwave Radiometer (MWR) in estimating both CAPE and CIN. MWR tended to underestimate CAPE and overestimate CIN by several hundred J/kg. CAPE estimates improved further when AERI data were paired with water vapor measurements from active systems. The ALVICE and MPD LiDAR systems, located at separate observation sites (FP2 and FP3, respectively), captured different perspectives of the outflow events. Radiosonde data at each site served as reference “truth” data for individual comparisons. The integration of ALVICE Raman LiDAR data significantly reduced RMSE in CAPE estimates (242.8 J/kg) and increased the Pearson correlation coefficient by 0.14. MPD observations also improved CAPE estimates, though to a lesser extent (−47 RMSE, +0.03 r). For CIN, AERI standalone data produced slightly better estimates than AERI + ALVICE at FP2, whereas AERI + MPD slightly outperformed AERI standalone data at FP3. A detailed look at a broader dataset is needed to assess whether these differences are statistically significant or influenced by factors such as instrument placement, storm proximity, or sample size. Key findings from additional analyses include the following:

- Airborne DIAL (LASE) water vapor profiles are used to demonstrate the platform’s ability to resolve CAPE and CIN across a large study domain using observational data. Derived values reveal substantial spatial variability in CAPE and CIN across the study domain for the case studies, highlighting a spatio-temporal evolution consistent with the onset of the 15 July MCS.
- WRF model simulations showed improved CAPE/CIN estimations when assimilating consensus, radar, and LiDAR observations.
- Observation resolution analysis identified 200 m as an optimal balance between instrument limitations and the ability to resolve mesoscale atmospheric features for research applications. Vertical resolutions larger than 500 m were found insufficient for calculating convection parameters with this method, particularly close to the surface.

These results have important implications for future atmospheric monitoring and forecasting. The demonstrated value of integrated remote sensing systems discussed above underlines the importance of their use in developing more robust and accurate convective forecasting indices. While this work tested only two cases, more robust work that builds on these and compares the full, seasonal instrument performance of more cases should be performed. We believe such work will complement and capture CAPE/CIN values from operational radiosondes launched only twice daily.

## Figures and Tables

**Table 1:** Instrument specifications.

Instrument	Type	Vertical Resolution	Temporal Resolution
AERI ^1^	Passive	100+ m	8 min
LASE (airborne) DIAL ^2^	Active	30 m	3 min
MicroPulse (ground-based) DIAL ^3^	Active	75 m	30 s
Radiosonde ^4^	In Situ	20–500 m	
ALVICE ^5^	Active	30 m	30 s
Microwave Radiometer ^6^	Passive	50 m	2 min

^1^ Demirgian et al. 2005 [10]; Knuteson et al. 2004 [11], ^2^ Ferrare et al. 2016 [12], ^3^ Spuler et al. 2015 [13], ^4^ Fairless et al. 2021 [14], ^5^ Whiteman et al. 2016 [15]; ^6^ Demoz et al. 2016 [16].

**Table 2:** Earth Science and Applications from Space (ESAS) measurement requirements for PBL variables: temperature (T), water vapor (q), and PBL height (PBLH) adapted from Table 3-1 in [18]. Italicized descriptions of the science objectives are from Table 3.2 in [17]. Importance is identified as I = Important, VI = Very Important, and MI = Most Important.

Objective (Importance)	Variable	Horizontal Resolution	Vertical Resolution	Temporal Resolution
W-1a (MI) *Determine the effects of key boundary layer processes on weather, hydrological, and air quality forecasts at minutes to subseasonal time scales*	T, q profiles	20 km	200 m	3 h
	PBLH	20 km	100 m	3 h
W-2a (MI) *Improve the observed and modeled representation of natural, low-frequency modes of weather/climate variability (e.g., MJO, ENSO)*	T, q profiles	3–5 km	1 km	1–3 h
W-3a (VI) *Determine how spatial variability in surface characteristics modifies regional cycles of energy, water, and momentum (stress) … and observe total precipitation to an average accuracy of 15% over oceans and/or 25% over land and ice surfaces*	PBLH	5–10 km	10 m	1–2/day
W-4a (MI) *Measure the vertical motion within deep convection to within 1 m/s and heavy precipitation rates to within 1 mm/hour to improve model representation of extreme precipitation and to determine convective transport and redistribution of mass, moisture, momentum, and chemical species*	q profiles	1 km	500 m	15 m
W-10a (I) *Quantify the effects of clouds of all scales on radiative fluxes, including on the boundary layer evolution. Determine the structure, evolution, and physical/dynamical properties of clouds*	cloud fraction	200 m	--	--

**Table 3:** Data variables and derived values.

Instrument/Data	Temperature	Dewpoint	Pressure
AERI	✓	✓	✓
ALVICE (Raman LiDAR)	FP2 AERI used	q ^1^ → Td	✓
NCAR MPD (DIAL)	FP3 AERI used	(n/V) ^2^ → Td	✓
Microwave Radiometer	✓	RH ^3^ → Td	z → P
LASE DIAL (Airborne)	ERA5 used	q ^1^ → Td	z → P
ERA5 (Reanalysis)	✓	RH ^3^ → Td	✓

^1^ Water vapor mixing ratio [g/kg]; ^2^ number density [molecules/cm^3^]; ^3^ relative humidity [%].

**Figure A3:**
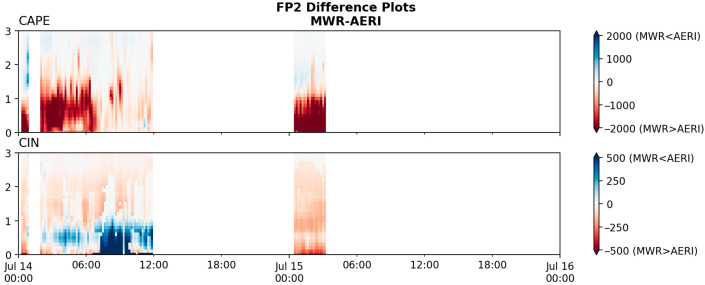
CAPE and CIN differences for FP2 AERI vs. MWR calculated values.

**Figure A4:**
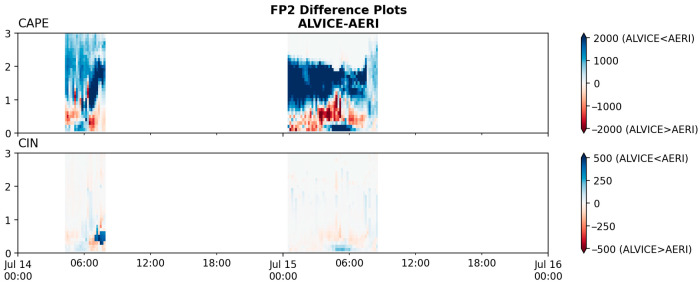
CAPE and CIN differences for FP2 AERI vs. ALVICE calculated values.

**Figure A5:**
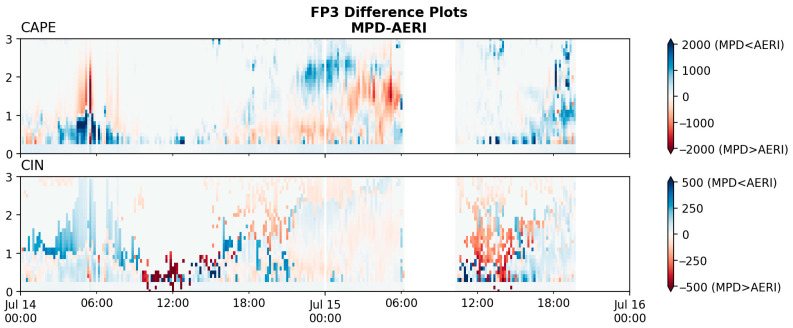
CAPE and CIN differences for FP3 AERI vs. MPD calculated values.

## Data Availability

Data are contained within the article.

## Abbreviations

The following abbreviations are used in this manuscript:

PBL	Planetary Boundary Layer
PECAN	Plains Elevated Convection At Night (Field Campaign)
MCS	Mesoscale Convective System
CI	Convection Initiation
CAPE	Convective Available Potential Energy
CIN	Convective Inhibition
DIAL	Differential Absorption LiDAR
MPD	MicroPulse DIAL
RMSE	Root Mean Squared Error
AERI	Atmospheric Emitted Radiance Interferometer
MWR	Microwave Radiometer
WRF	Weather Research and Forecasting (model)

## Appendix A

Data collected by the surface-based profilers AERI, MWR, ALVICE, and MPD and used in CAPE and CIN calculations are shown in Figure A1 and Figure A2 below. Radiosonde profiles are overlaid between white lines for reference.

## Appendix B

Difference plots of calculated CAPE and CIN are shown in Figure A3 and Figure A4 to illustrate magnitude and sign of difference between collocated data products.
